# Development and Validation of Genome Instability-Associated lncRNAs to Predict Prognosis and Immunotherapy of Patients With Hepatocellular Carcinoma

**DOI:** 10.3389/fgene.2021.763281

**Published:** 2022-01-28

**Authors:** Yifeng Yan, Liang Ren, Yan Liu, Liang Liu

**Affiliations:** ^1^ Department of Forensic Medicine, Tongji Medical College, Huazhong University of Science and Technology, Wuhan, China; ^2^ Department of Forensic Medicine, Wannan Medical College, Wuhu, China

**Keywords:** hepatocellular carcinoma, TCGA, GILncSig, TP53, immunotherapy

## Abstract

The pathophysiology of hepatocellular carcinoma (HCC) is prevalently related to genomic instability. However, research on the association of extensive genome instability lncRNA (GILnc) with the prognosis and immunotherapy of HCC remains scarce. We placed the top 25% of somatic mutations into the genetically unstable group and placed the bottom 25% of somatic mutations into the genetically stable group, and then to identify different expression of GILnc between the two groups. Then, LASSO was used to identify the most powerful prognostic GILnc, and a risk score for each patient was calculated according to the formula. Based on a computational frame, 245 different GILncs in HCC were identified. An eight GILnc model was successfully established to predict overall survival in HCC patients based on LASSO, then we divided HCC patients into high-risk and low-risk groups, and a significantly shorter overall survival in the high-risk group was observed compared to those in the low-risk group, and this was validated in GSE76427 and Tongji cohorts. GSEA revealed that the high-risk group was more likely to be enriched in cancer-specific pathways. Besides, the GILnc signature has greater prognostic significance than TP53 mutation status alone, and it is capable of identifying intermediate subtype groups existing with partial TP53 functionality in TP53 wild-type patients. Importantly, the high-risk group was associated with the therapeutic efficacy of PD-L1 blockade, suggesting that the development of potential drugs targeting these GILnc could aid the clinical benefits of immunotherapy. Finally, the GILnc signature model is better than the prediction performance of two recently published lncRNA signatures. In summary, we applied bioinformatics approaches to suggest that an eight GILnc model could serve as prognostic biomarkers to provide a novel direction to explore the pathogenesis of HCC.

## Introduction

Hepatocellular carcinoma (HCC) is the most common cause of death in patients with chronic liver disease, and the fourth most common cause of death from cancer (Siegel et al., 2020; Eloranta et al., 2021). Its recurrence rate is up to 70%, and the 5-year survival rate is less than 60% with successful surgical resection or liver transplantation. Despite achieving great progress in the prevention, diagnosis, and treatment of this disease in recent years, the clinical outcome is still unsatisfactory (Forner et al., 2018; Villanueva, 2019). Hence, the identification of a novel and reliable prognostic molecular signature to screen appropriated therapeutic strategies or improve unfavorable prognosis is urgently needed for HCC cohorts.

Molecular biology studies have shown that there is genomic instability or genetic instability in HCC (Dore et al., 2001). Loss of heterozygosity (LOH) and microsatellite instability (MSI) caused by DNA mismatch repair (MMR) gene repair errors are considered to be the two main phenotypic features of genome instability (Tubbs and Nussenzweig, 2017). Genomic stability can be a hallmark of both human genetic disease and cancer (Andor et al., 2017). The relative stability of the genome is the basic prerequisite for cells to be faithfully passaged. When there are genetic defects or exposure to adverse environmental factors such as biological, physical, and chemical tests, it will lead to genomic instability (Gerlach and Herranz, 2020). Studies have constructed 10-miRNA signatures related to DNA damage response, and have shown that 10-miRNA signatures are associated with poor prognoses of ovarian cancer (Wang et al., 2017). More and more evidence indicates that lncRNA plays an important role in tumors, and abnormal lncRNA expression may affect tumor cell proliferation, tumor progression or metastasis (Zhang et al., 1993; Luo et al., 2006; Sanchez Calle et al., 2018). Therefore, constructing lncRNAs related to genomic instability may be a prognostic factor for HCC.

In this study, we download HCC patients from TCGA and GSE76427, and a systematic analysis was conducted on genome instability lncRNA signature (GILncSig) to screen different expression genes (DEGs) in HCC. After univariate and multivariate Cox regression analysis, eight GILncSigs were screened to establish the prognostic risk score model to evaluate the prognosis of HCC. Predicting genes were validated in testing set. Finally, GSE76427 was used to compare the expression level of eight GILncSigs between HCC tissues and the paired adjacent normal tissue (PANT).

## Materials and Methods

### Data Acquisition

RNA-seq datasets, as well as corresponding clinical information, were downloaded from the TCGA database (https://cancergenome.nih.gov/) and processed through the R package “TCGA-Assembler.” A total of 424 HCC patients (normal = 50, tumor = 374) were enrolled in our study from the TCGA dataset. GSE76427 (normal = 52, tumor = 115) was download from the GEO database (http://www.ncbi.nih.gov/geo) (Grinchuk et al., 2018). We divided all TCGA HCC samples into a training set and a test set. The training set included 184 samples for the creation of a clinical outcome lncRNA risk model. The test set included 181 patients and was used to validate the predictive ability of the prognostic risk model. Meanwhile, the somatic mutation information data was downloaded from TCGA (Supplementary Table S1). The detailed clinical characteristics of these patients are summarized in Table 1.

**TABLE 1:** Clinical characteristics of HCC patient datasets in this study.

Characteristic		TCGA dataset (*N* = 365)	Training dataset (*N* = 184)	Testing dataset (*N* = 181)	*p*-value
Age (years), n (%)	≤65	227 (62.2%)	120 (65.2%)	107 (59.1%)	0.274
	>65	138 (37.8%)	64 (34.8%)	74 (40.9%)	
Gender, *n* (%)	Female	119 (32.6%)	60 (32.6%)	59 (32.6%)	1.000
	Male	246 (67.4%)	124 (67.4%)	122 (67.4%)	
Grade, *n* (%)	G1-2	230 (63.0%)	110 (59.8%)	120 (66.3%)	0.259
	G3-4	130 (35.6%)	71 (38.6%)	59 (32.6%)	
	unknow	5 (1.4%)	3 (1.6%)	2 (1.1%)	
Stage, *n* (%)	Stage I/II	254 (69.6%)	117 (64.6%)	137 (74.5%)	0.014
	Stage III/IV	87 (23.8%)	54 (29.8%)	33 (17.9%)	
	unknow	24 (6.6%)	10 (5.5%)	14 (7.6%)	
T, *n* (%)	T1-2	271 (74.3%)	125 (69.1%)	146 (79.4%)	0.020
	T3-4	92 (25.2%)	56 (30.9%)	36 (19.6%)	
	unknow	2 (0.6%)	0 (0%)	2 (1.1%)	
M, *n* (%)	M0	263 (72.6%)	131 (72.4%)	132 (71.7%)	1.000
	M1	3 (0.8%)	2 (1.1%)	1 (0.5%)	
	unknow	99 (27.1%)	48 (26.5%)	51 (27.7%)	
*N*, *n* (%)	N0	248 (68.0%)	120 (66.3%)	128 (69.6%)	0.581
	N1-3	4 (1.1%)	3 (1.7%)	1 (0.5%)	
	unknow	113 (31.0%)	58 (32.0%)	55 (29.9%)	

### Selection of GILncSig in HCC

To identify genome instability-associated lncRNAs, a mutator hypothesis-derived computational frame combining lncRNA expression profiles and somatic mutation profiles in a tumor genome was developed: 1) the cumulative number of somatic mutations for each patient was computed; 2) patients were ranked in decreasing order of the cumulative number of somatic mutations; 3) the top 25% of patients were defined as genomic unstable (GU)-like group, and the last 25% were defined genomically stable (GS)-like group; 4) expression profiles of lncRNAs between the GU group and GS group were compared using significance analysis of microarrays (SAM) method; 5) differentially expressed lncRNAs (false discovery rate adjusted *p* < 0.05) were defined as genome instability-associated lncRNAs.

Limma packages in R language were used to screen the differentially expressed GILncSig in HCC. The default Benjamini-Hochberg FDR was employed to adjust the *p*-value to remove false positive results. Differently expressed GILncSig were processed by hierarchical clustering analysis with R package “pheatmap” (Robinson and Oshlack, 2010).

### Functional Enrichment Analysis

Gene set enrichment analysis (GSEA) was processed by GSEA software version 3.0. used to enrich key signaling pathways. We computed the Pearson correlation coefficients to measure the correlation between the paired expression of lncRNAs and mRNAs, and the top 10 mRNAs were considered as co-expressed lncRNA-associated partners. To predict the potential functions of lncRNAs, we performed functional enrichment analysis of co-expressed lncRNA-associated mRNA partners to determine significantly enriched GSEA Gene Ontology (GO) terms and GSEA Kyoto Encyclopedia of Genes and Genomes (KEGG) pathway. The functional enrichment analysis was performed using clusterProfiler software in R-version 3.5.2. FDR <0.25 and |ES| >0.8 were the cutoff criteria to decide a statistical significance (Carlevaro-Fita et al., 2019).

### Construction of a Risk Model With Prognostic Value in HCC

Hierarchical cluster analyses were performed using Euclidean distances and Ward’s linkage method. Univariate and multivariate Cox proportional hazard regression analysis was used to evaluate the association between the expression level of genome instability-associated lncRNA and overall survival. Based on the coefficients from the multivariate regression analysis and the expression levels of prognostic genome instability-associated lncRNAs, we constructed a genome instability-derived lncRNA signature (GILncSig) for outcome prediction as follows:

$$GILncSig\;Riskscore=\sum_{i=1}^{n}coef\left(IncRNAi\right)*\exp r\left(IncRNAi\right)$$

where GILncSig (patient) is a prognostic risk score for the breast cancer patient, lncRNAi represents the *i*th prognostic lncRNA, expr (lncRNAi) is the expression level of lncRNAi for the patient, and coef (lncRNAi) represents the contribution of lncRNAi to prognostic risk scores that were obtained from the regression coefficient of multivariate Cox analysis. The median score of the patients in the training set was used as a risk cutoff to classify patients into the high-risk group with high GILncSig or low-risk group with low GILncSig (Tibshirani, 1997; Simon et al., 2011).

### Assessment of Immune Cells Infiltration

We performed the ssGSEA method to calculate the enrichment scores on the basis of metagenes. The reason why we considered the metagene a robust approach was the two main characteristics: 1) the use of a set of genes instead of single genes that represent one immune subpopulation because the use of single genes as markers for immune subpopulations can be misleading as many genes are expressed in different cell types; and 2) the assessment of relative expression changes of a set of genes in relation to the expression of all other genes in a sample. Referring to the Bindea et al. study, the author incorporate 535 metagenes that represented diverse 24 immune subpopulations: innate immune cells (dendritic cells [DCs], immature DCs [iDCs], activated DCs [aDCs], plasmacytoid DCs [pDCs], eosinophils, mast cells, macrophages, natural killer cells [NKs], NK CD56dim cells, NK CD56bright cells, and neutrophils) and adaptive immune cells (B cells, T cells, T helper cells, T gamma delta [Tgd] cells, T helper 1 [Th1] cells, Th2 cells, Th17 cells, regulatory T [Treg] cells, CD8^+^ T cells, T central memory [Tcm] cells, T effector memory [Tem] cells, T follicular helper [Tfh] cells, and cytotoxic cells).

### Quantitative Reverse Transcription Polymerase Chain Reaction (qRT-PCR) Assays

A total of 150 HCC patients’ tissues and corresponding adjacent tissues were collected to explore the expression of eight GILncs in the tissue samples by using qRT-PCR, which was performed according to the manufacturer’s instructions.

### Statistical Analyses

The normality of the variables was evaluated using the Shapiro-Wilk normality test. Kaplan-Meier analysis was used to generate survival curves using the “survival” and “survminer” packages, and the cut-off values were determined through the “surv_cutpoint” function in the packages. To calculate the hazard ratios and identify the independent prognostic factors, univariate and multivariate Cox regression analyses were performed using the “survival” package. Subsequently, we employed the area under the ROC curve (AUC) to measure the prediction accuracy. All statistical analyses were two-sided and considered *p* < 0.05 as the threshold for statistical significance. The statistical results were all analyzed by R (version3.6.2).

## Result

### Identification of GILncSigs in HCC Patients

**FIGURE 1:**
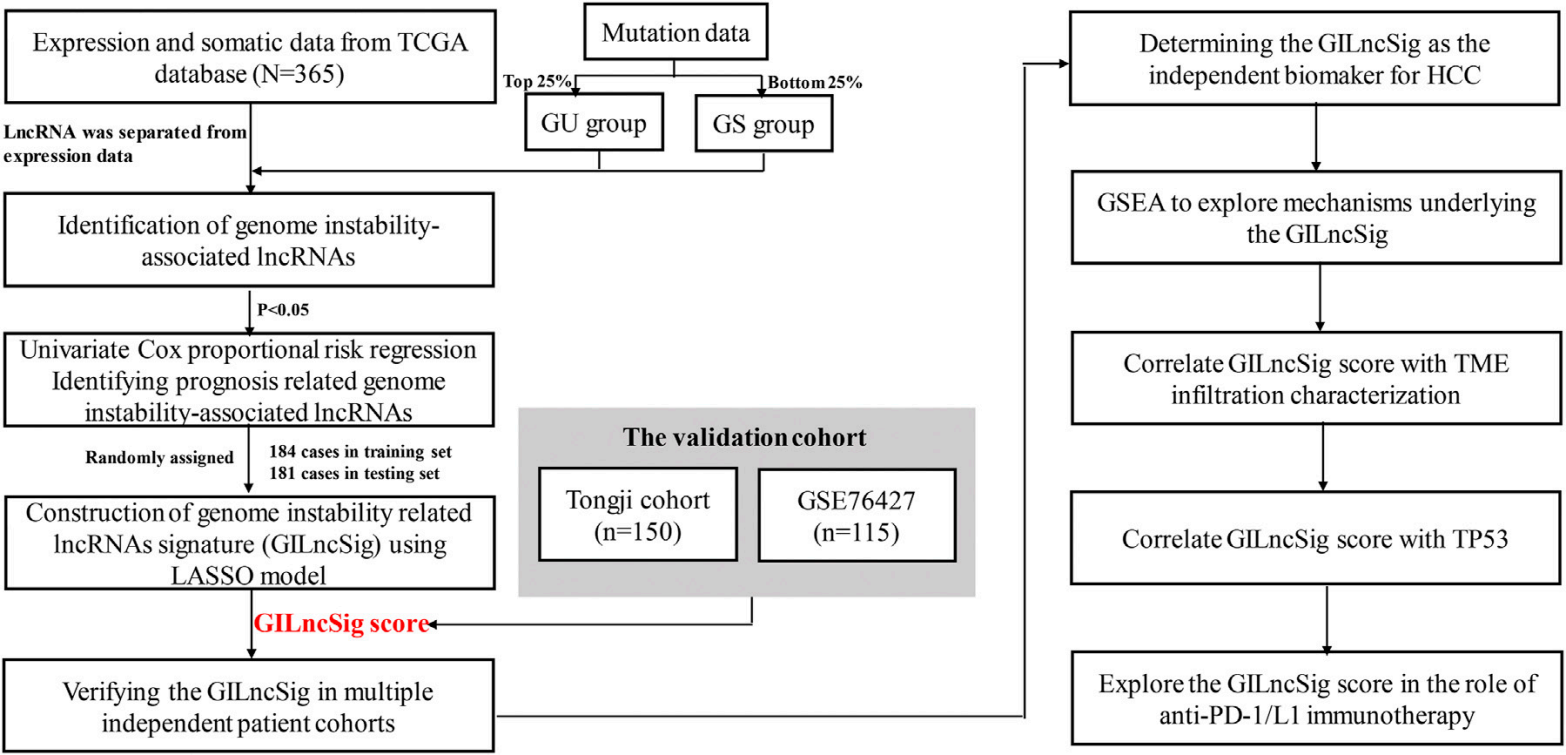
Flow chart of the steps in the performed analyses.

**FIGURE 2:**
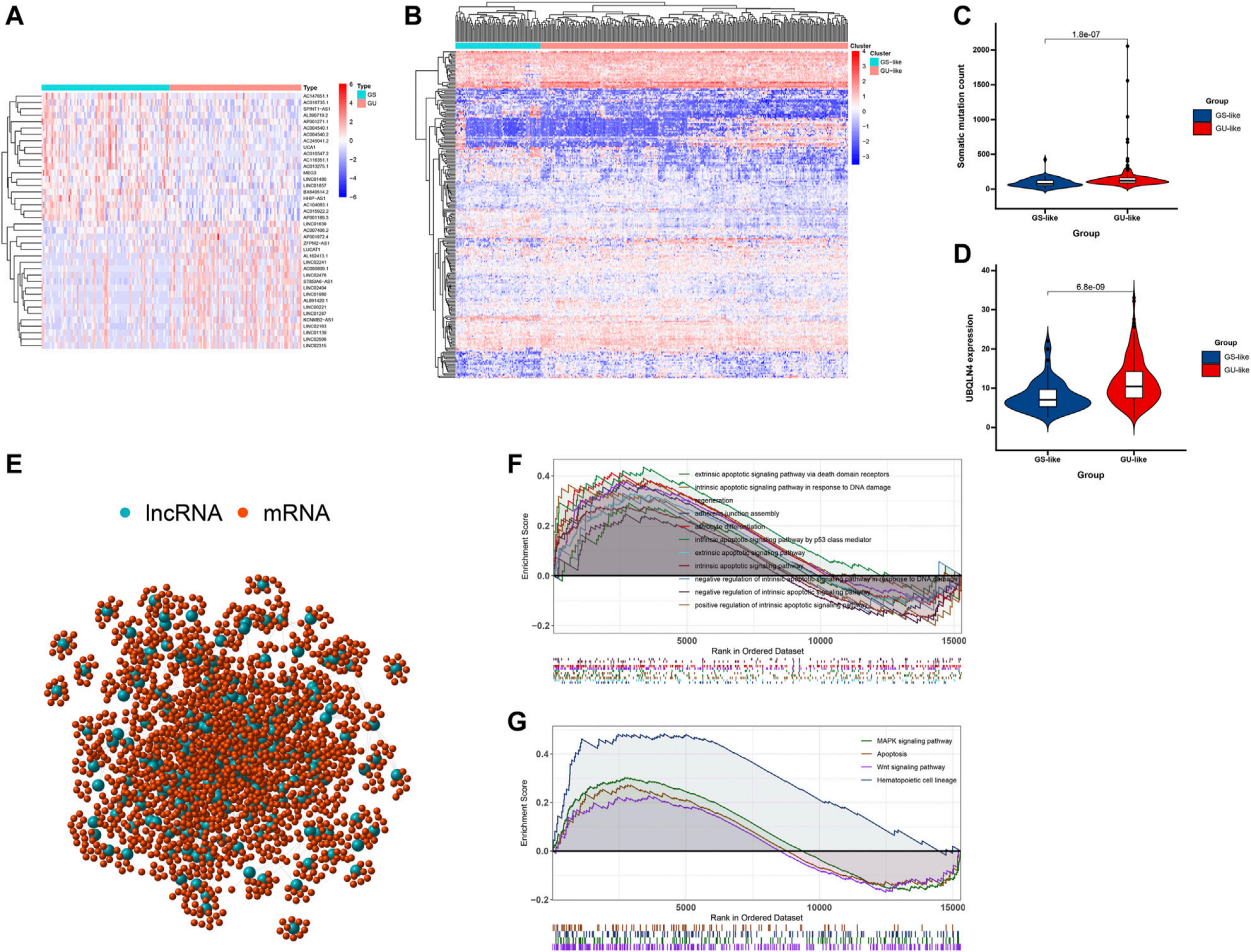
Identification and functional annotations of genomic instability-related lncRNAs in patients with breast cancer. **(A)** Heatmap of the top 20 genome instability-associated lncRNAs expressing the most upregulation and downregulation. **(B)** Unsupervised clustering of 374 HCC patients based on the expression pattern of 245 candidate genomic instability-related lncRNAs. The left blue cluster is GS-like group, and the right red cluster is GU-like group. **(C)** Boxplots of somatic mutations in the GU-like group and GS-like group. Somatic cumulative mutations in the GU-like group are significantly higher than those in the GS-like group. **(D)** Boxplots of UBQLN4 expression level in the GU-like group and GS-like group. The expression level of UBQLN4 in the GU-like group is significantly higher than that in the GS-like group. Horizontal lines: median values. Statistical analysis was performed using the Mann–Whitney U test. **(E)** Coexpression network of genomic instability-related lncRNAs and mRNAs based on the Pearson correlation coefficient. The red circles represent lncRNAs, and the blue circles represent mRNAs. **(F)** Functional enrichment analysis of GESA GO for mRNAs co-expressed lncRNAs. **(G)** Functional enrichment analysis of GESA KEGG for mRNAs co-expressed lncRNAs.

The workflow of our study was shown in Figure 1. To identify the GILncSigs in HCC, the top 25% somatic mutations per patient (*n* = 93) and the least 25% somatic mutations per patient (*n* = 90) of the patients were assigned to GU-like (genomic unstable) group and GS-like (genomic stable) group. As shown in Supplementary Table S2, 245 GILncSigs were significantly different in expression between the GU-like group and the GS-like group (Figure 2A). Using 245 differentially expressed GILncSigs, all 374 patients were arranged into two clusters, the GS-like cluster and the GU-like cluster (Figure 2B). Next, we further analyzed the difference in the cumulative somatic mutation value between the GU group and the GS group, and the results showed that the cumulative somatic mutation value of the GU group was significantly higher than that of the GS group (Figure 2C). Researchers have discovered UBQLN4 is a newly identified driver of genomic instability, and harmful UBQLN4 mutations occur in families with autosomal recessive syndromes. Loss of UBQLN4 can lead to increased sensitivity to genotoxic stress and delayed DNA double-strand break (DSB) repair (Jachimowicz et al., 2019). Therefore, we compared the expression level of UBQLN4 in the GU group and the GS group, and the results showed that the expression level of UBQLN4 in the GU group was significantly higher than that in the GS group (Figure 2D).

To better understand the functions of these GILncSigs, we constructed a lncRNA-mRNA co-expression network, where the nodes are lncRNA and mRNA, if they are related to each other, lncRNA and mRNA will be linked together (Figure 2E). In addition, functional enrichment analysis of GESA for lncRNA-correlated mRNA, GSEA showed that lncRNA-correlated mRNA are mainly cancer-specific pathways (Figures 2F,G).

### Development of a GILncSig for Prognosis of Patients With HCC in the Training Set

**FIGURE 3:**
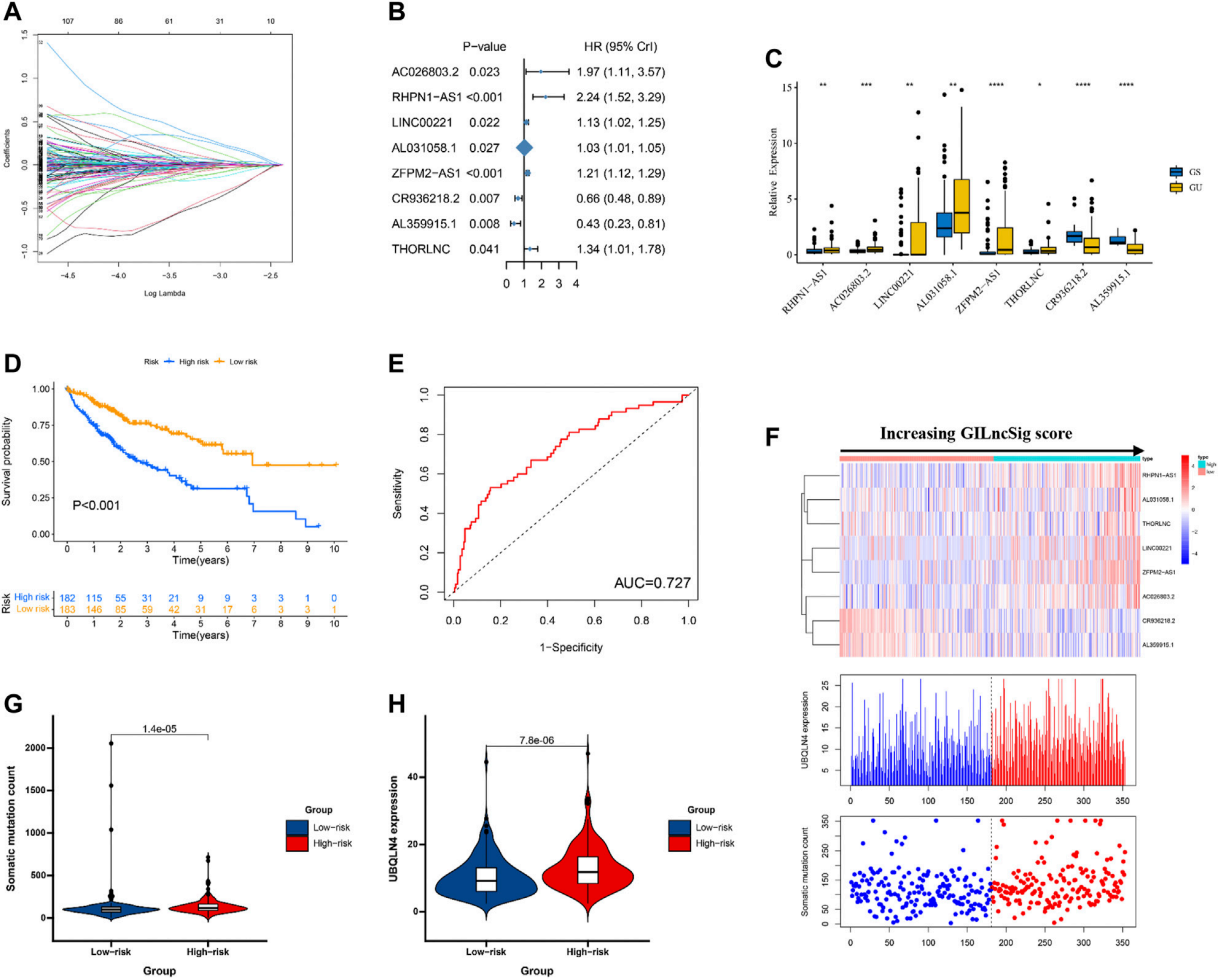
Identification of the genomic instability-derived lncRNA signature (GILncSig) for outcome prediction in the TCGA set. **(A)** The coefficients calculated by multivariate Cox regression using LASSO are shown. **(B)** Forest plots showing the results of the multivariate Cox regression between GILncSig expression and OS. **(C)** The expression level of 8 GILncs in GS and GU groups. **(D)** Kaplan–Meier estimates of overall survival of patients with low or high risk predicted by the GILncSig in the TCGA set. Statistical analysis was performed using the log-rank test and univariate Cox analysis. **(E)** Time-dependent ROC curves analysis of the GILncSig at 1 year **(F)** LncRNA expression patterns and the distribution of somatic mutation and UBQLN4 expression with increasing GILncSig score. The distribution of somatic cumulative mutations **(G)** and UBQLN4 expression in the **(H)** in the high- and low-risk groups for HCC patients. The red represents the high-risk group, and the blue represents the low-risk group. Horizontal lines: median values. Statistical analysis was performed using the Mann–Whitney U test.

**FIGURE 4:**
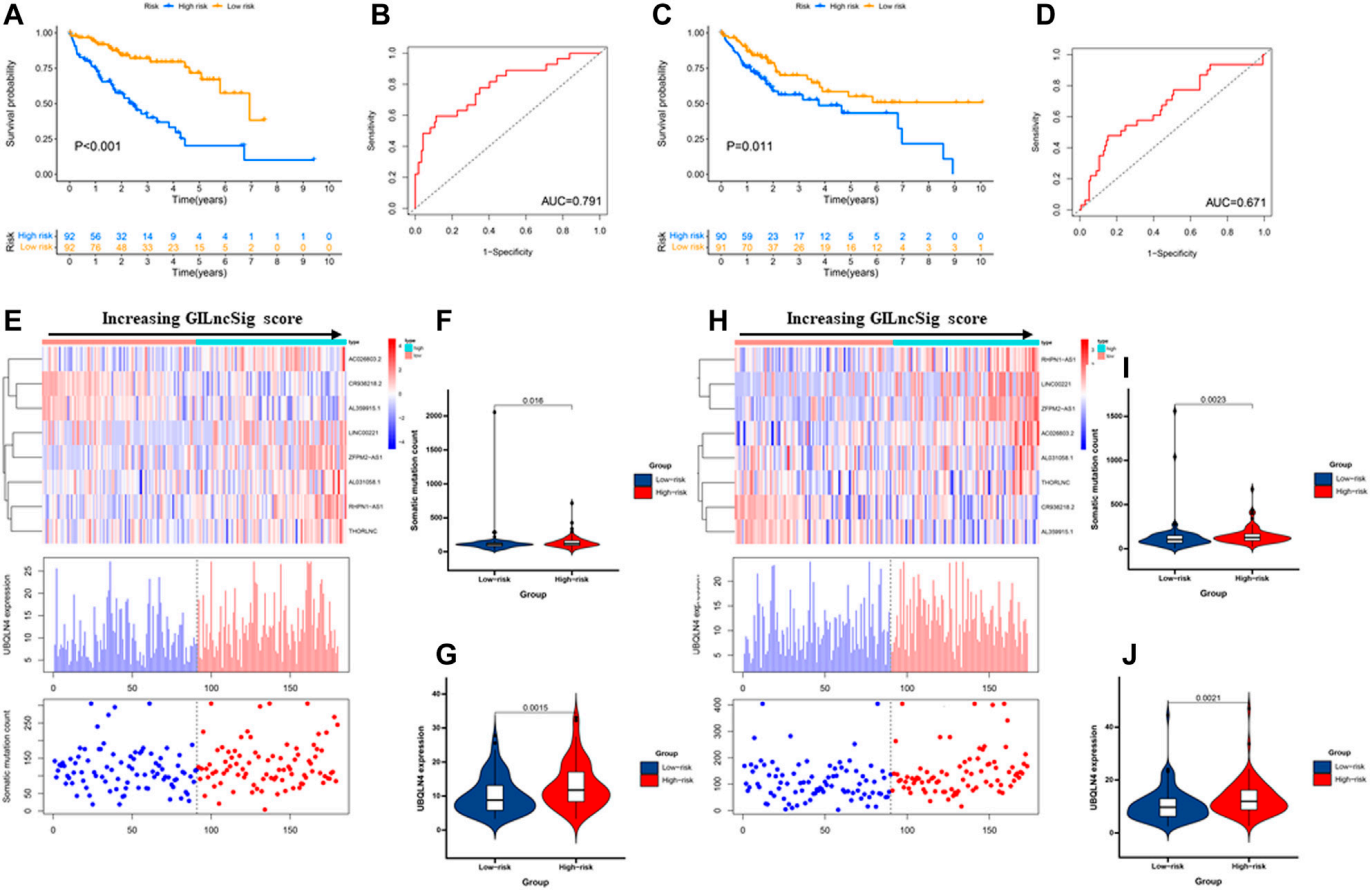
Performance evaluation of the GILncSig in the training set and testing set. Kaplan–Meier estimates of overall survival of patients with low or high risk predicted by the GILncSig in the training set **(A)** and testing set **(C)**. Statistical analysis was performed using the log-rank test and univariate Cox analysis. Time-dependent ROC curves analysis of the GILncSig at 1 year in the training set **(B)** and testing set **(D)**. LncRNA expression patterns and the distribution of somatic mutation count distribution and UBQLN4 expression for patients in high- and low-risk groups in the training set **(E)** and testing set **(H)**. The distribution of somatic mutation in patients of high- and low-risk groups in the training set **(F)** and testing set **(I)**. The distribution of UBQLN4 expression in patients of high- and low-risk groups in the training set **(G)** and testing set **(J)**. Horizontal lines: median values. Statistical analysis was performed using the Mann–Whitney U test.

Next, 374 HCC patients were divided into a training set and a testing set according to best batches. In order to predict the clinical outcomes of HCC with GILncSig, we applied the least absolute shrinkage and selection operator (LASSO) Cox regression algorithm to the 245 GILncSigs in the training set. Eight GILncSigs were selected to build the risk signature based on the minimum criteria, and the coefficients obtained from the LASSO algorithm were used to calculate the risk score for training set (Figure 3A). Next, to inspect whether the eight GILncSigs were related to prognosis in HCC, multivariate Cox regression was employed to analyze the hazard ratio (HR) of the eight GILncSigs in HCC. Forest plotting showed that high expression levels of six genes including AC026803.2, RHPN1-AS1, LINC00221, AL031058.1, ZFPM2-AS1, and THORLNC were significantly related to poor overall survival (OS) of HCC patients. Meanwhile, high expression levels of CR936218.2 and AL359915.1 were closely related to relative better OS in HCC patients (Figure 3B; Table 2). Compared with the GS group, the AC026803.2, RHPN1-AS1, LINC00221, AL031058.1, ZFPM2-AS1, and THORLNC level were significantly higher in the HCC group, however, the CR936218.2 and AL359915.1 levels were significantly lower in the HCC group (Figure 3C). The training set patients were assigned to low-risk and high-risk groups based on the median value of risk scores, and patients in the high-risk group had poor survival compared with the low-risk group (Figure 4A). To test the efficiency of GILncSig, ROC curve was constructed. The risk score’s AUC was 0.791, indicating that its efficiency to predict prognosis was accurate (Figure 4B). Simultaneously, as increasing GILncSig score, the change of expression in the training set (Figure 4E). We further analyzed the difference between the cumulative somatic mutation value between the two groups, and the cumulative somatic mutation value of the high-risk group was significantly higher than that of the low-risk group (Figure 4F). We also compared the expression level of UBQLN4 in the two groups, and the expression level of UBQLN4 in the high-risk group was significantly higher than that in the low-risk group (Figure 4G).

**TABLE 2:** Multivariate Cox regression analysis of genome instability-related lncRNAs associated with overall survival in HCC.

Ensembl ID	Gene symbol	Genomic location	Coefficient	HR	95% CI	*p*-value
ENSG00000267898	AC026803.2	Chromosome 19: 48,963,975-48,965,158	1.08	1.97	1.11−3.57	0.023
ENSG00000254389	RHPN1-AS1	Chromosome 8: 143,366,631-143,368,548	0.62	2.24	1.52−3.29	<0.001
ENSG00000270816	LINC00221	Chromosome 14: 106,482,435-106,521,073	0.09	1.13	1.02−1.25	0.022
ENSG00000261189	AL031058.1	Chromosome 6: 7,540,451-7,541,338	0.03	1.03	1.01−1.05	0.027
ENSG00000251003	ZFPM2-AS1	Chromosome 8: 105,546,089-106,060,524	-1.544	1.21	1.12−1.29	<0.001
ENSG00000262881	CR936218.2	Chromosome 17: 45,907,670-45,910,779	-0.905	0.66	0.48−0.89	0.007
ENSG00000227712	AL359915.1	Chromosome 1: 119,230,313-119,327,915	0.404	0.43	0.2−-0.81	0.008
ENSG00000226856	THORLNC	Chromosome 2: 118,132,128-118,222,250	0.198	1.34	1.01−1.78	0.041

### Validation of a GILncSig for Outcome Prediction in the Testing Set and TCGA Set

**FIGURE 5:**
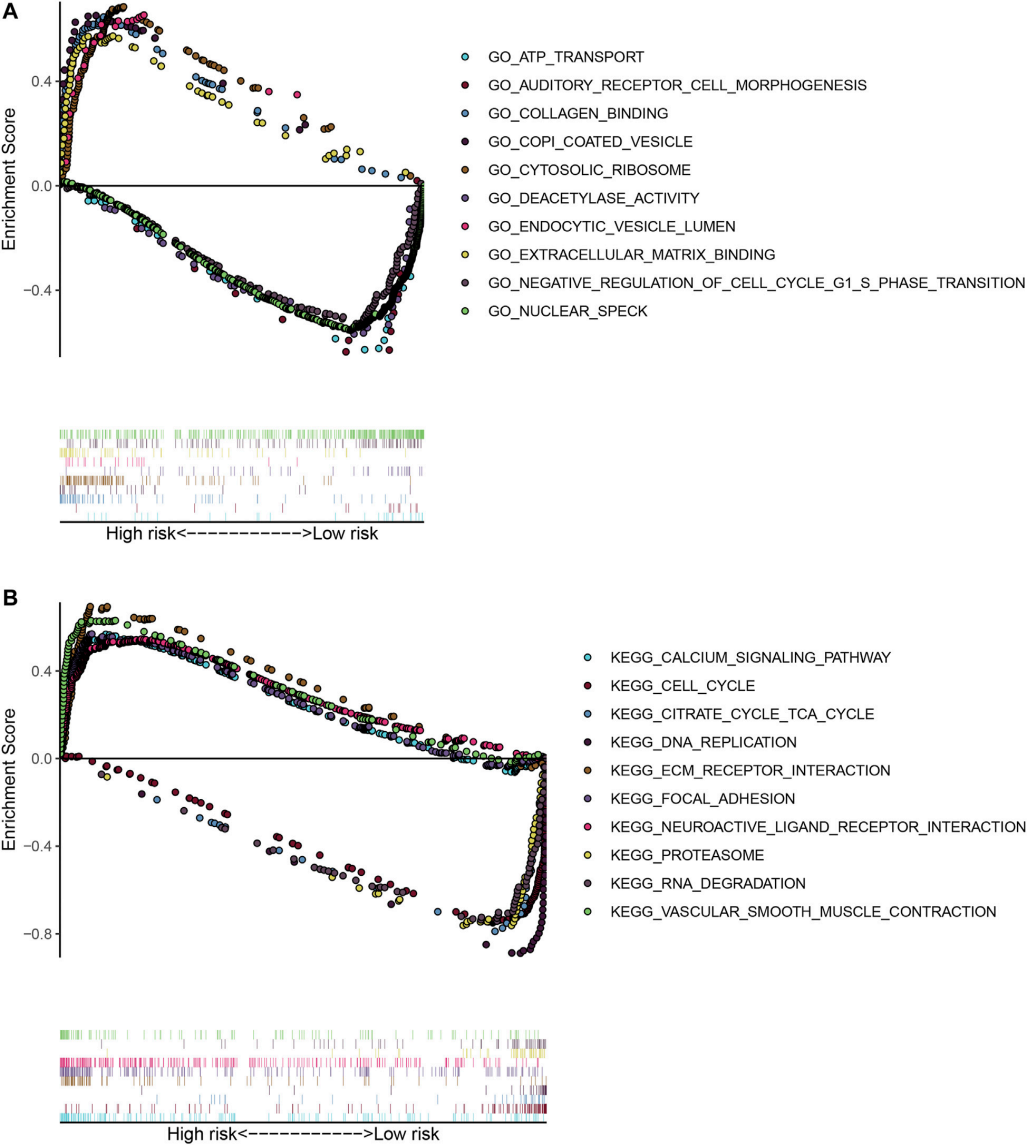
GSEA on the TCGA cohort to explore mechanisms underlying the 8- GILncSig. **(A)** GSEA GO identifies high- and low-risk related signaling pathways in HCC. **(B)** GSEA KEGG identifies high- and low-risk related signaling pathways in HCC.

To examine the robustness of the GILncSig in the testing set and TCGA set. The results showed that patients in the high-risk group had poor survival compared with the low-risk group in the testing set and TCGA set (Figures 3D, 4C). The risk score’s AUC was 0.727 in the TCGA set and 0.671 in the testing set, indicating that its efficiency to predict prognosis was accurate (Figures 3E, 4D). Simultaneously, as increasing GILncSig score, the change of expression in the testing set and TCGA set (Figures 3F, 4H). We further analyzed the difference in the cumulative somatic mutation value between the two groups, and the results showed that the cumulative somatic mutation value of the high-risk group was significantly higher than that of the low-risk group in the testing set and TCGA set (Figures 3G, 4J). We also compared the expression level of UBQLN4 in the two groups in the testing set and TCGA set, and the expression level of UBQLN4 in the high-risk group was significantly higher than that in the low-risk group (Figures 3H, 4J). To explore the functional role of this prognostic signature in HCC progression, we performed GSEA analysis, and the results indicated that the high-risk group was more likely to be enriched in cancer-specific pathways (Figures 5A,B). The above-mentioned evidence demonstrated that the prognostic signature based on eight GILncSigs is reliable in HCC prognosis.

### Independent Prognostic Analysis of GILncSig

**FIGURE 6:**
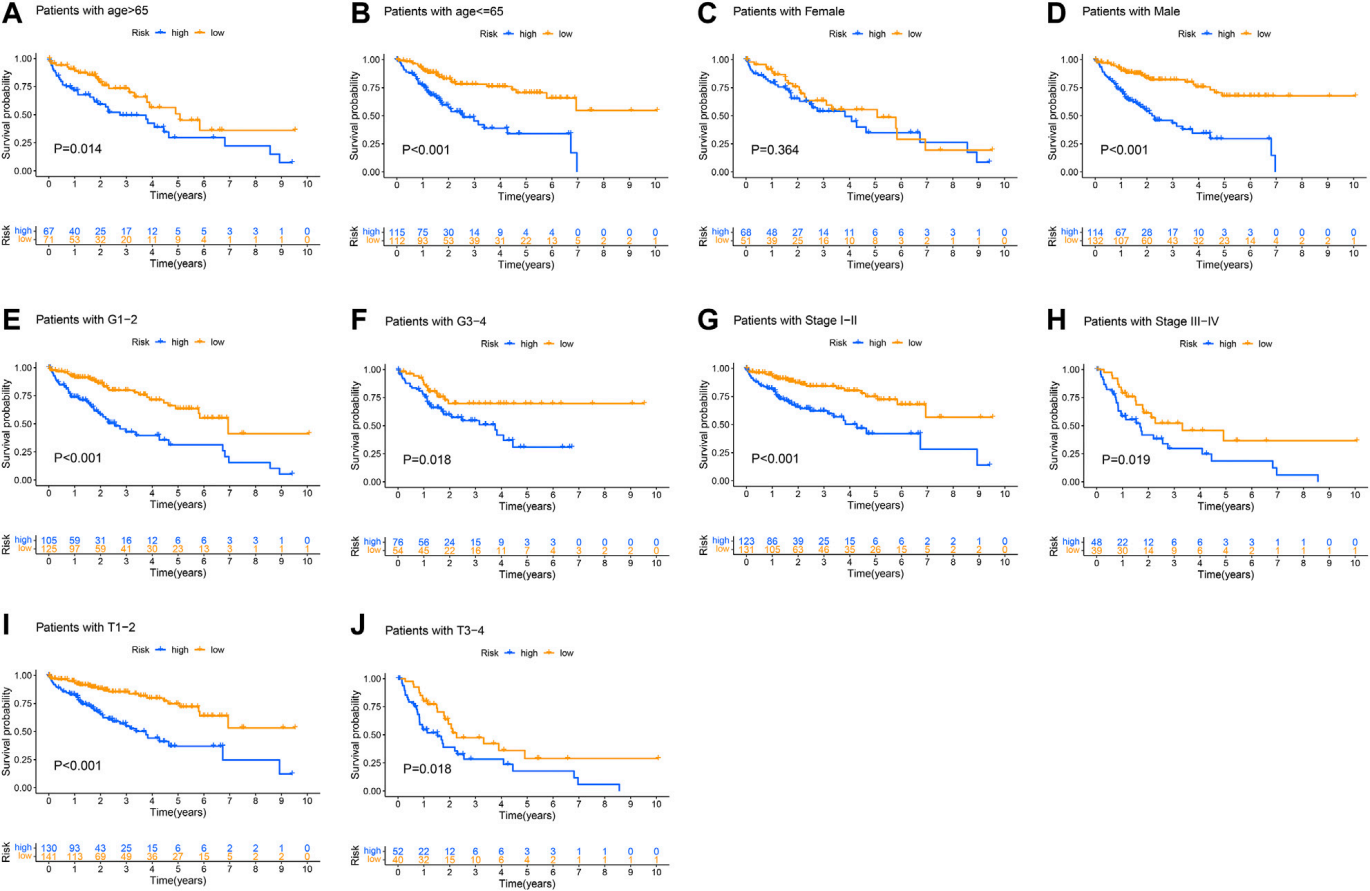
Kaplan–Meier curves were performed for patients stratified by clinicopathological features in the TCGA set. Impact of prognostic risk on overall survival for patients younger than 65 years old **(A)** and older than 65 years old **(B)**; for female **(C)** and male patients **(D)**; for patients in G1-2 **(E)** and G3-4 **(F)**; for patients in stage I-II **(G)** and stage III-IV **(H)**; and for patients in stage T1-2 **(I)** and stage T3-4 **(J)**.

**FIGURE 7:**
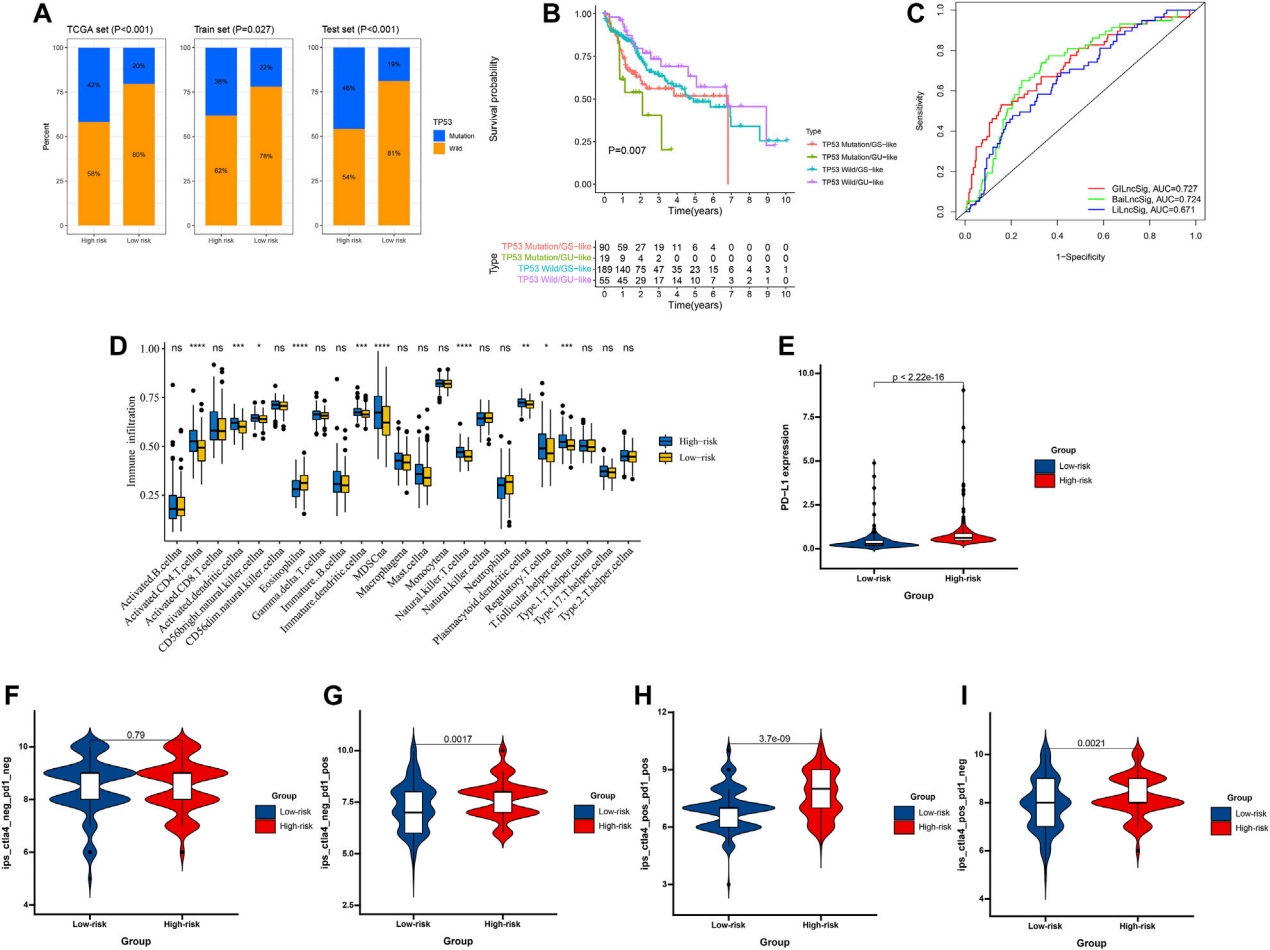
Relationship between the GILncSig and TP53 somatic mutation. **(A)** The proportion of TP53 mutation in high- and low-risk groups in the training set, testing set, and the TCGA set. **(B)** Kaplan–Meier curve analysis of overall survival is shown for patients classified according to TP53 mutation status and the GILncSig. Statistical analysis was performed using the log-rank test. **(C)** The ROC analysis at 3 years of overall survival for the GILncSig, LilncSig, and BailncSig. **(D)** The abundance of each TME infiltrating cell in high-risk and low-risk groups. The upper and lower ends of the boxes represented the interquartile range of values. The lines in the boxes represented median value, and black dots showed outliers. The asterisks represented the statistical *p* value. **(E)** PD-L1 expression patterns for patients in high-risk and low-risk groups in the TCGA cohort. The correlation of GILncSig score with clinical response to anti-PD-1 immunotherapy. **(F)** CTLA4_negative + PD-1_negative, **(G)** CTLA4_negative + PD-1_positive, **(H)** CTLA4_positive + PD-1_positive, **(I)** CTLA4_positive + PD-1_negative.

Next, multivariate Cox regression analysis was performed on the clinical factors and GIlncSig risk score to determine the independent prognostic value of GilncSig (Table 3). As shown in Figures 6A,B, in both the young and old groups, the survival rate of the high-risk group was significantly lower than that of the low-risk group (*p* < 0.05). Subsequently, the survival rate of the high-risk group was significantly lower than that of the low-risk group in the pathological stage (Figures 6E–J). However, there were different results in male and female patients (Figures 6C,D). Finally, we compared the resulting GIlncSig to the latest published signatures related to lncRNAs; the first signature is the 4-lncRNA signature (BailncSig), and the second signature is the 3-lncRNA signature (LilncSig). The results showed that the AUCs for BailncSig (Bai et al., 2021), LilncSig (Li et al., 2021), and GILncSig OS were 0.724, 0.671, and 0.727 (Figure 7C). Based on the above results, GlncSig has independent research value in HCC.

**TABLE 3:** Univariate and multivariate Cox regression to identify independent prognosis predictor in both the TCGA cohort and the GSE76427 cohort.

Characteristics	TCGA cohort				GSE76427 cohort			
	Univariate mode		Multivariate model		Univariate mode		Multivariate model	
	HR (95% CI)	*p*-value	HR (95% CI)	*p*-value	HR (95% CI)	*p*-value	HR (95% CI)	*p*-value
Age (≥60 vs. < 60)	1.02 (0.98–1.03)	0.154	0.99 (0.93–1.09)	0.358	1.17 (0.80–1.72)	0.315	1.52 (0.84–2.48)	0.530
Gender (Male vs. Female)	0.88 (0.68–1.13)	0.188	0.90 (0.61–1.33)	0.594	1.06 (0.60–1.75)	0.828	NA	NA
AFP (≥200 vs. < 200)	0.92 (0.60–1.36)	0.724	0.80 (0.21–1.87)	0.932	NA	NA	NA	NA
TMB (TMB-H vs TMB-L)	**1.20 (1.07–1.32)**	**<0.001**	**1.11 (1.02–1.20)**	**<0.001**	NA	NA	NA	NA
Tumor grade (G3/4 vs. G1/2)	1.23 (0.98–1.46)	0.331	1.10 (0.92–1.46)	0.483	NA	NA	NA	NA
Tumor stage (III/IV vs. I/II)	1.01 (0.99–1.05)	0.363	0.99 (0.85–1.25)	0.462	1.39 (0.70–3.51)	0.495	NA	NA
Vascular invasion (Yes vs. No)	1.83 (0.74–3.96)	0.375	1.15 0.62–2.73)	0.625	NA	NA	NA	NA
Risk (High vs. Low)	**1.15 (1.10–1.20)**	**<0.001**	**1.13 (1.07–1.19)**	**<0.001**	**1.72 (1.15–2.43)**	**0.023**	**1.61 (1.04–2.21)**	**0.035**

p value less than 0.05 is considered as significance with bold fonts.

### GILncSig is Better Than TP53 Mutation Status in Predicting HCC Prognosis

It has been confirmed that the abnormality of the TP53 gene is closely related to the occurrence and development of HCC (Hill et al., 2018). As shown in Figure 7A, the proportion of patients with TP53 mutations in the high-risk group was significantly higher than that in the low-risk group (*p* < 0.05). Next, to evaluate whether GILncSig is better than TP53 mutation status predicting HCC prognosis, we divided all patients into TP53 mutation/GS, TP53 mutation/GU, TP53 wild/GS, and TP53 wild/GU groups. The results showed that patients in the TP53 mutation/GU group had poor survival as compared with the TP53 mutation/GS group in the TCGA set (Figure 7B).

### The GILncSig in the Role of PD-1/L1 Immunotherapy

HCC is immunogenic and immunosuppressed, thus, the application of immunotherapy is of great significance for the development of THE treatment of HCC. It has been confirmed that the tumor microenvironment (TME) is correlated with the sensitivity to immunotherapy and HCC prognosis, and the ssGSEA method was used to calculate the enrichment scores on the basis of metagenes, and was employed in the gene expression data from three datasets to calculate an immune score. As shown in Figure 7D, the proportion of immune cells in the high-risk group was significantly lower than that in the low-risk group (*p* < 0.05), suggesting that the immune ability of the high-risk group is suppressed. Then, we analyzed the expression of PD-L1 between the high-risk and low-risk groups, and significantly higher PD-L1 expression in the high-risk group was observed relative to the low-risk group (Figure 7E). Next, we download the immunotherapy data of TCGA-LIHC patients, and the significant therapeutic advantages and clinical response to PD-1/L1 immunotherapy in patients in the high-risk group compared to those in the low-risk group were confirmed (Figures 7F–I).

### Validation of the Expression and Prognosis of GILncSig in GSE76427 and Tongji Cohorts

**FIGURE 8:**
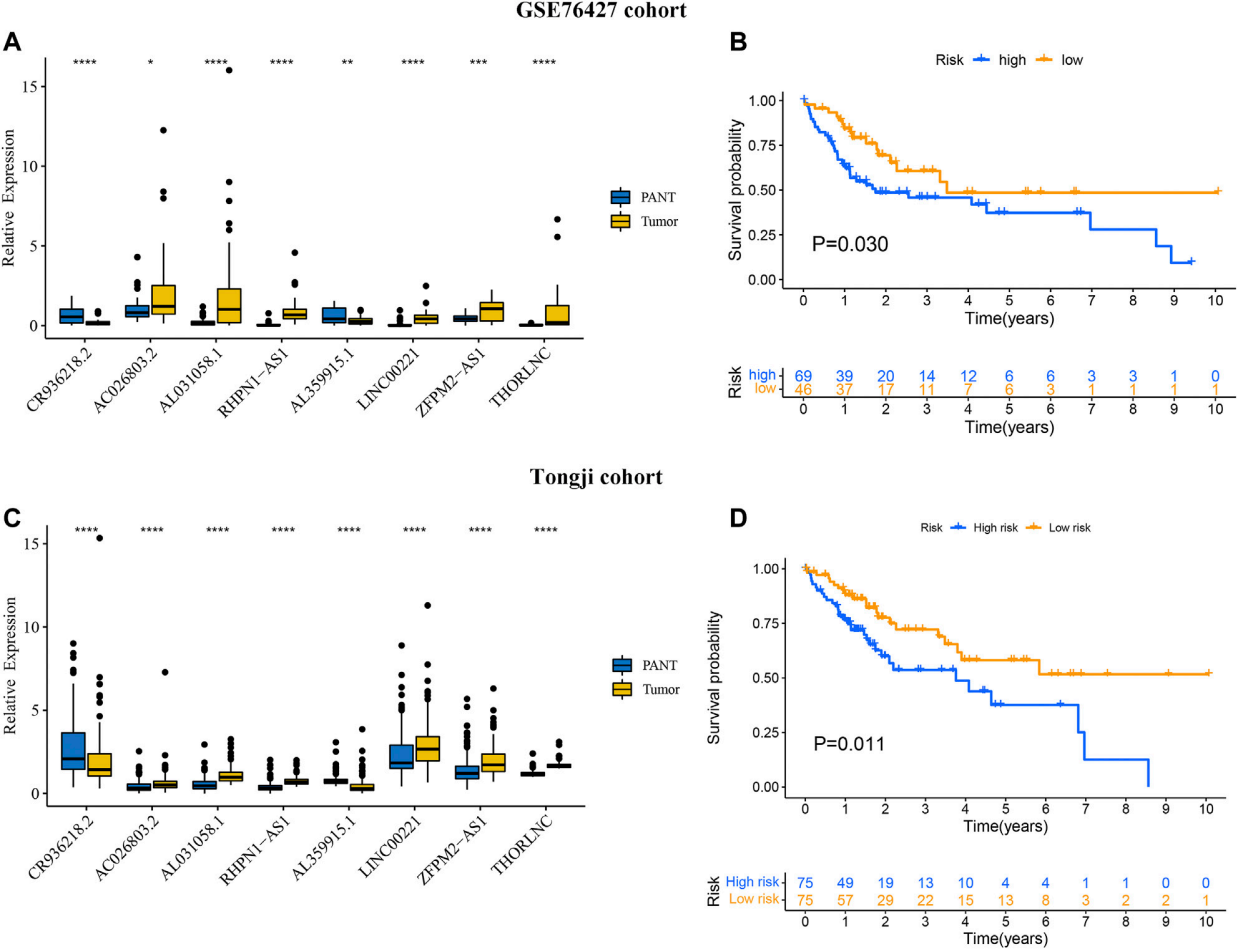
Validation of the prognostic performance of GIlncSig on GSE76427 and Tongji datasets. The expression level of eight GILncs for the HCC and paired adjacent normal tissue (PANT) in GSE76427 **(A)** and Tongji datasets **(C)**. Kaplan–Meier curves illustrated that patients with high risk had worse overall survival than those with low risk in GSE76427 **(B)** and Tongji datasets **(D)**.

To further validate GILncSig expression in HCC, GSE76427 was used to measure the expression level of GILncSig, and the results showed that compared with PANT group, the AC026803.2, RHPN1-AS1, LINC00221, AL031058.1, ZFPM2-AS1, and THORLNC levels were significantly higher in the HCC group, however, the CR936218.2 and AL359915.1 levels were significantly lower in the HCC group (Figures 8A,C). In addition, base on the 8-GILncSig, patients in the high-risk group had poor survival as compared with the low-risk group (Figures 8B,D).

## Discussion

The occurrence of a malignant tumor is a process of multi-gene participation and gradual evolution (Hanahan and Weinberg, 2011). The progression of a normal cell to a malignant cell is actually a long process that involves various genetic mutations that lead to a precancerous lesion and then to a malignant tumor. This series of genomic evolution often requires DNA damage or replication abnormalities to chromosomal instability and even the emergence of a “hyperploidy” phenotype. As a result, most tumors often present a complex genetic map at the time of diagnosis, which is very different from that of normal controls, suggesting a high degree of genomic instability in the body at the time of tumor development (Sieber et al., 2003; Pikor et al., 2013). Genomic instability is an important molecular feature of malignancy (Abbas et al., 2013). The relative stability of the genome is the basic prerequisite for faithful cell passage (Ovejero et al., 2020). Detection of genomic instability is now thought to be an early warning of tumorigenesis (Sieber et al., 2005). Current studies have also confirmed that genes that cause genomic instability are important clues to the causes of tumors (Petropoulos et al., 2019). Moreover, with the continuous improvement of modern molecular biology methods, more and more evidence shows that cancer patients can be timely treated by detecting their genomic instability (Ben-David et al., 2019). Clinically, there have been some targeted drugs targeting genomic instability-related genes, which have brought good news to tumor patients (O'Shaughnessy et al., 2011). It has been established that downregulation of these genomic instability-related genes has been clinically found to significantly enhance the sensitivity of cancer patients to platinum-based chemotherapy (Ledermann et al., 2012; Simmons et al., 2016). To sum up, it is of great significance to elucidate the relevant mechanisms of genomic instability in tumor cells and to conduct relevant assays to reduce the incidence of tumor, delay the progression, and improve the disease condition.

LncRNA is widely defined as a class of RNA molecules that have a transcriptional length is greater than 200 nucleotides and lack an open reading frame (Esteller, 2011). LncRNAs have a potential role in regulating the function of tumor cells (Bhan et al., 2017). LncRNA regulates gene expression at different levels including chromatin assembly, transcriptional, and posttranscriptional (Mercer et al., 2009; Schmitt and Chang, 2016). Recent studies have shown that NORAD and GUARDIN are essential for genomic stability (Hu et al., 2018; Munschauer et al., 2018). Currently, a variety of tumor genomic instability detection technologies have sprung up rapidly, and the understanding of the role of genomic instability in the development of tumors is gradually deepening. However, the genom-wide identification of genomic instability-related lncRNAs and the systematic exploration of their clinical significance in cancer are still in their infancy. Therefore, it is of great significance to identify lncRNAs associated with genomic instability.

First, we downloaded the TCGA-LiHC expression profile data and mutation data. We defined the 25% with the highest frequency as the high mutation group, and the 25% with the lowest frequency as the low mutation group. A total of 245 different lncRNAs were obtained compared with the low mutation group. These lncRNAs were characterized as genomic instability-related lncRNAs. Functional enrichment analysis of mRNAs co-expressed with 245 lncRNAs indicated that these lncRNAs may play an important role in the pathogenesis, DNA damage, and apoptosis pathways of HCC, which is consistent with other studies (Gillman et al., 2021). Abnormal repair of DNA damage is directly related to genomic stability (Tubbs and Nussenzweig, 2017). If the mechanism of repairing DNA damage is defective, it will directly lead to the persistence of DNA damage and the harmful changes of cells, until the tumor is triggered. DNA damage is mainly exogenous, such as chemical exposure, UV irradiation, biological hazards, and endogenous, such as *in vivo* spontaneous DNA damage events, cell cycle process and DNA replication process block. These damages, if not repaired in time, can induce genomic oxidation, alkylation, and even DNA crosslinking, dimer formation and even DNA breakage. Therefore, whether DNA damage can be repaired in time and correctly directly affects the maintenance of genome stability (Pommier et al., 2016; De Magis et al., 2019; Fang and Ding, 2020). Next, we constructed 8-GILncSig to predict the course of HCC patients. GILncSig divided patients into low-risk and high-risk groups. The mutation frequency of the high-risk group was higher than that of the low-risk group in both the training set and the test set. In addition, similar results were found in the GSE76427 dataset. Furthermore, we compared the resulting GIlncSig to the latest published signatures related to lncRNAs; the first signature is the 4-lncRNA signature (BailncSig), and the second signature is the 3-lncRNA signature (LilncSig). The results showed that GILncSig is superior to the other two models.

Among the eight GILncs, we found that three lncRNAs (RHPN1-AS1, LINC00221, and ZFPM2-AS1) have been reported to be related to HCC. RHPN1-AS1 was upregulated in HCC tissue, and silencing lncRNA RHPN1-AS1 also inhibited the activation of PI3K/AKT/mTOR pathway (Song et al., 2020). Human HCC samples had increased the expression of LINC00221. LINC00221 knockdown repressed HCC cell growth, migration, and invasion and enhanced their apoptosis (Yang et al., 2021). ZFPM2-AS1 was observed to be distinctly upregulated in HCC tissues and associated with shorter overall survival. Inhibition of ZFPM2-AS1 suppressed cell proliferation, metastasis, cell cycle progression while accelerated cell apoptosis (Zhang et al., 2021).

There are several limitations to this study. First, there is a lack of biological verification. Future molecular studies are needed to identify the interactions between the eight GIlncSigs from our prognostic model. Moreover, all data in this study is obtained from public databases, so future studies with prospective validation are still warranted.

## Conclusion

In conclusion, this work identified and validated an 8-GIlncSig model that can predict the outcome of HCC. This prognostic model can be a promising tool for predicting the clinical prognosis and guiding strategies for HCC, which also provides a wider insight for understanding the HCC.

## Data Availability

The original contributions presented in the study are included in the article/Supplementary Material, further inquiries can be directed to the corresponding author.
